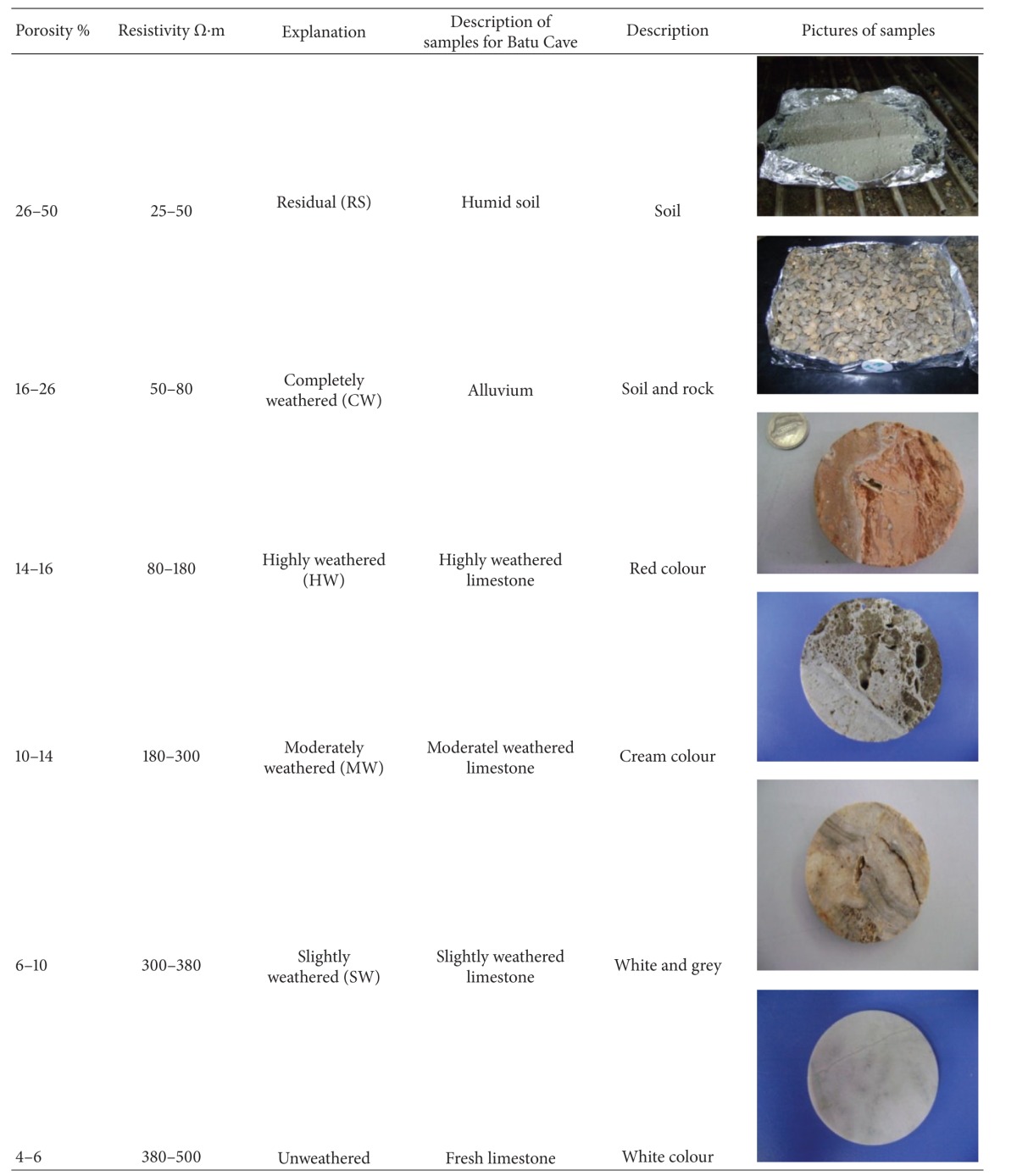# Application of Geophysical Techniques for 3D Geohazard Mapping to Delineate Cavities and Potential Sinkholes in the Northern Part of Kuala Lumpur, Malaysia

**DOI:** 10.1155/2013/629476

**Published:** 2013-12-31

**Authors:** Zeinab Bakhshipour, Bujang B. K. Huat, Shaharin Ibrahim, Afshin Asadi, Nura Umar Kura

**Affiliations:** ^1^Civil Engineering Department, Faculty of Engineering, University Putra Malaysia, 43400 Serdang, Selangor, Malaysia; ^2^Faculty of Environmental Studies, Universiti Putra Malaysia (UPM), 43400 Serdang, Selangor, Malaysia; ^3^Housing Research Center, Civil Engineering Department, Faculty of Engineering, University Putra Malaysia, 43400 Serdang, Selangor, Malaysia

## Abstract

This work describes the application of the electrical resistivity (ER) method to delineating subsurface structures and cavities in Kuala Lumpur Limestone within the Batu Cave area of Selangor Darul Ehsan, Malaysia. In all, 17 ER profiles were measured by using a Wenner electrode configuration with 2 m spacing. The field survey was accompanied by laboratory work, which involves taking resistivity measurements of rock, soil, and water samples taken from the field to obtain the formation factor. The relationship between resistivity and the formation factor and porosity for all the samples was established. The porosity values were plotted and contoured. A 2-dimensional and 3-dimensional representation of the subsurface topography of the area was prepared through use of commercial computer software. The results show the presence of cavities and sinkholes in some parts of the study area. This work could help engineers and environmental managers by providing the information necessary to produce a sustainable management plan in order to prevent catastrophic collapses of structures and other related geohazard problems.

## 1. Introduction

Karst areas are known to have a unique set of geotechnical and environmental difficulties that affects land use [1]. Irrespective of whether karst structures are exposed or not, they pose serious threats to properties such as buildings, agricultural farmland, roads, and railways. An example of karst-related destruction is the collapse of a highway bridge over the Seti River [2, 3]. Numerous engineering problems are believed to be connected with construction in karst environments, such as the disastrous collapse of the ground surface or a slow unnoticeable subsidence, which among other things, could lead eventually to the collapse of buildings, the destruction of railways and roads due to subsidence, and dam failures. The formation of large voids in areas underlain by carbonate rocks may lead to either a gradual ground subsidence due to the slow migration of fine particles from the subbase or to a sudden and catastrophic pavement failure, such as a sinkhole [1, 4].

Damage related to sinkholes is not limited to properties and structures such as buildings and roads but it also affects water and environmental resources, creating pathways for draining surface water such as streams and lakes, directly into the underlying aquifers. Furthermore, this leads to the contamination of groundwater through the transportation of pollutants into the aquifer [5].

The process of karst formation commences as rainfall (H_2_O) passes from the atmosphere onto the top soil, where it then infiltrates the ground. Mixed with (CO_2_) gas from the air and soil, this water produces weak carbonic acid (H_2_CO_3_), which seeps further into the ground and makes contact with the limestone (CaCO_3_) and/or dolomite (CaMg (CO_3_)_3_) [5]. This leads to the dissolution of these rocks and the development of the karst, which is characterised by voids and cavities, sinkholes, sinking streams, and the presence of irregular rock surfaces with soil-filled lots and pinnacles [6]. Therefore, prior to any activity of development in an area with sinkholes and karst formations, there is the need for a comprehensive management plan to prevent sinkhole-related disasters. One of the most important measures mitigating the development of sinkholes is to understand and control the surface and groundwater systems of areas underlain by carbonate rocks. These problems are usually addressed through different types of engineering technique, for example, grouting; however, the effects of such techniques on groundwater flow are usually not considered [1].

A major challenge facing researchers and engineers in assessing karsts is the identification and delineation of underground cavities. These structures are usually unpredictable and their effects can either lead to a slow and gradual subsidence or to a catastrophic collapse feature [1]. Geological and geomorphological methods are some of the techniques used in tackling these problems but a major limitation regarding these techniques is that they are not applicable in urban areas, particularly those areas experiencing rapid development, owing to the concealment of surface features by building structures and other development activities. Moreover, karst systems and structures represent disturbances of the close subsurface layered system within these areas and thus hazard mapping, especially for civil engineering purposes, is impossible with limited sources of information [4]. Therefore, the need to understand how karstic systems function involves the use of a number of indirect techniques that rely on the interpretation of hydrodynamic and other related methods combined with geophysics. This allows engineers and researchers to assess the extent of karstification and the quantity of groundwater resources within the area, which enables them to have a clearer understanding of the geometry and structure of the karst system [7].

Geophysical methods such as seismic, microgravity, self-potential, electrical resistivity, electromagnetic, and ground-penetrating radar are more advantageous for geohazard investigations because of their nonintrusive nature and cost effectiveness [4, 8, 9]. This is particularly the case in urbanised areas, in which the use of direct methods is virtually impossible [10]. Therefore, the application of geophysical techniques for the assessment of karst features has gained wide recognition during the past few decades [4, 8].

The detection of the precise locations of voids is a major challenge but at the same time, it is a matter of necessity for risk assessment of collapse and for avoiding any destruction associated with soil degradation. This problem becomes increasingly complex in areas where the presence of natural cavities is not known [11]. In such areas, geophysical methods can provide a cost-effective solution for investigating the subsurface and for detecting cavity formation and voids [4, 11, 12]. In this context, the principal task of geophysical surveys is to identify precisely the presence of cavities [9].

However, it is not always easy to choose the correct tool for geophysical detection of subsurface cavities and sinkholes because of the uncertainty related to the characteristics of the target [4]. Many studies have demonstrated strong correlation between geophysical signals and known karstic voids; however, investigating for unknown karst features is still one of the most difficult tasks facing geophysicists [4, 8, 13, 14]. Therefore, further analysis, such as the determination of the formation factor and porosity, would not only increase the validity of the results but would also decrease the uncertainty in data interpretation.

This work attempts to establish a relationship between porosity and the resistivity of soil and rock samples to develop a better understanding of subsurface resistivity distribution via 2D electrical resistivity profiles. In order to convert the resistivity image into a geological image and subsurface porosity, knowledge both of the typical resistivity values for various types of subsurface materials and of the geology of the area in question is necessary. The resistivity of rocks and soils varies widely. This paper characterises the relationship between resistivity and soil and rock samples, derived through laboratory methods, for developing the geometry of cavities and sinkholes using 2D and 3D electrical resistivity (ER) techniques.

### 1.1. Study Area

**Figure 1:**
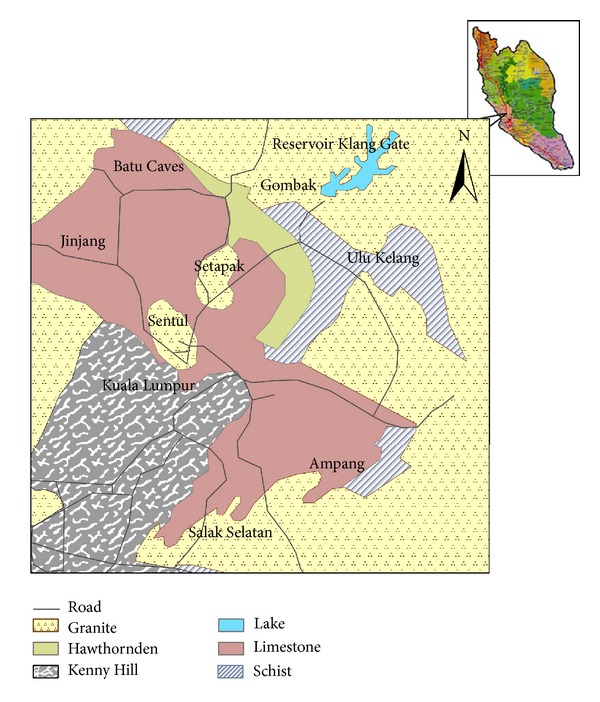
Geological map of the study area [15].

**Figure 2:**
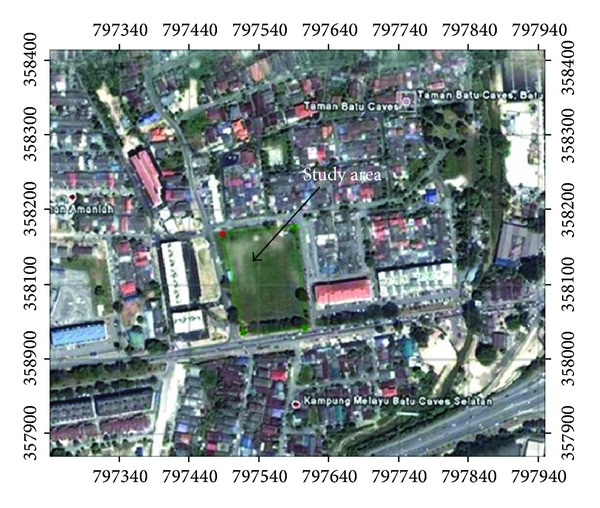
Study area of Batu Cave (Google Map, 2011).

The relatively flat study area (Figures 1 and 2) is located at latitude 3°14.4′N and longitude 101°41.2′E, which is approximately 300 m south of the Batu Cave limestone hill in Kuala Lumpur. A survey by the Department of Minerals and Geoscience, Malaysia (previously called: Malaysian Department of geological survey), revealed three geological layers in the area. The first is thin humid soil (approximately 0.37 m thick), followed by an alluvial stratum. The final layer portrays light grey limestone bedrock, which consists of many cavities [15]. It has been estimated that nearly 40% of Kuala Lumpur (236.827 Km^2^) is dominated by limestone [16]; thus, the limestone of this area is part of the Kuala Lumpur limestone formation [15]. The limestone underwent a dolomitisation process through chemical substitution, thermal metamorphism, and recrystallisation by thermal solutions of silica (SiO_2_) leading to the formation of coarse and fine crystal marble [16].

### 1.2. Materials and Method

#### 1.2.1. Field and Laboratory Studies

**Figure 3:**
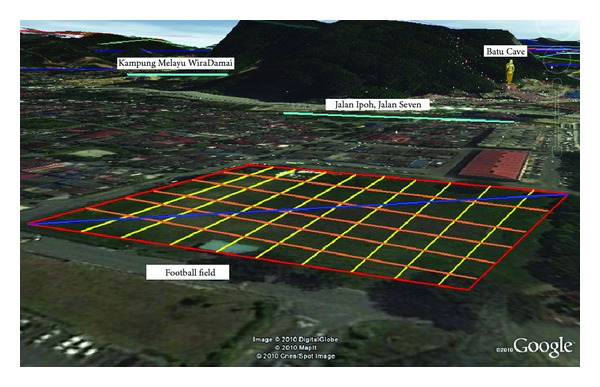
Map from Google Earth showing alignment of resistivity lines on the study area and two additional lines: one near the river and the other near Batu Cave Mountain.

An ABEM Terrameter SAS 1000/4000 was used to perform the electrical resistivity measurements in the field, utilising the Wenner configuration, because of its ability to detect vertical changes, horizontal structures, and strong signal strengths [17]. In all, 17 profile lines, each 80 m long, were measured with an electrode spacing of 2 m intervals designed to coincide with the dimensions of the studied field. The resistivity data obtained from the field were then inverted using RES2DINV software via apparent resistivity to obtain the true resistivity and true depth of the resistivity image. The lines were arranged to create a grid (Figure 3) of the entire area to acquire as much detailed information as possible. Nine of these lines (1–9) were oriented East-West and seven lines (11–17) were aligned North-South. One profile (line 10) was run diagonally (Figure 3). The length of each line is 80 m with the exception of the diagonal line, which is 160 m.

**Figure 4:**
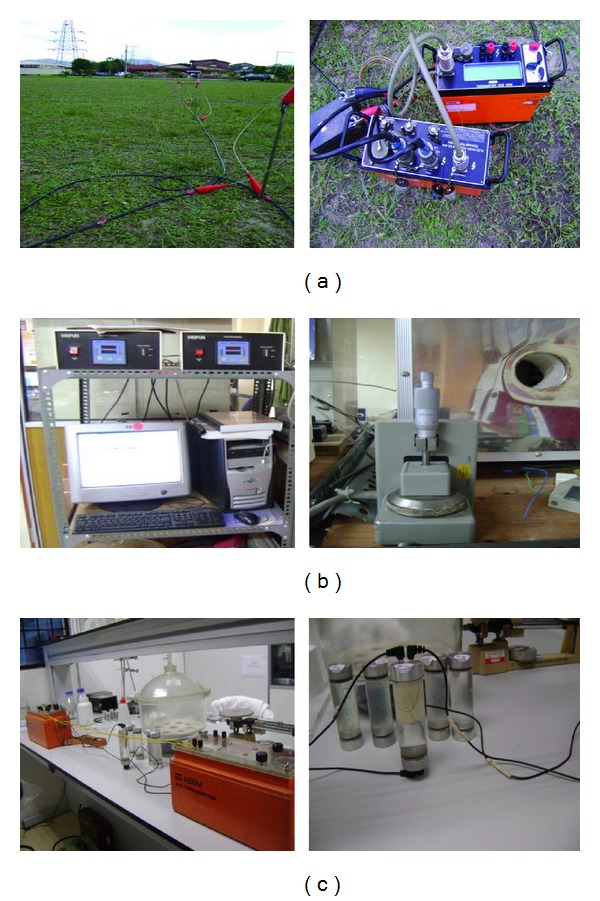
Field and laboratory measurement of electrical resistivity. (a) ABEM Terrameter (Ohm/Ω) resistivity meter in the field (Model-2115), (b) a precision inductance, conductance, and resistance (LCR) meter for conductance and resistance of rock (LCR), and (c) ABEM Terrameter (Ohm/Ω) resistivity meter of soil and water in laboratory.

Soil and core rock samples were collected along the resistivity profile lines. The soil samples were collected from top soil to the depth of one (m). Each sample underwent laboratory measurements of physical properties, such as the effective porosity, particle size distribution, bulk density, moisture content, and resistivity. The apparatus used for both field and laboratory analyses are shown in Figures 4(a), 4(b), and 4(c).

#### 1.2.2. Estimation of Effective Porosity and Formation Factor

The study of porosity and its distribution is very important because of its relationship with other geophysical parameters, such as resistivity and the associated formation factor. Effective porosity as defined by [18] as a segment of the soil or rock that contributes to flow. Mathematically, it is the ratio of the interconnectivity of pore volume to the total volume of the medium. Based on these definitions, effective porosity maintains the water in the formation and accounts for the space they occupy; thus, it better represents the formation.

For a rock saturated with water, Archie [19] established an experimental relationship linking the resistivity of the rock, the porosity, the nature of the distribution, and the resistivity of the electrolyte as follows:

(1) $$\begin{array}{c} R_{\text{rock}}=R_{w}\alpha \varphi^{-m}, \end{array}$$

where *R*_rock_ is the bulk resistivity of the rock in (Ω·m), *R*_*w*_ is the resistivity of the formation water in (Ω·m), *φ* is the porosity (%), *m* is the cementation factor, and *α* is associated with the porous medium.

The purpose of determining the effective porosity and the formation factor is to establish a relationship between the equations established in the laboratory from integrated soil and rock samples and 2D ER obtained from the field to derive the subsurface distribution of porosity and other associated features of the area under investigation.

In this work, the ER images were used to delineate and locate the various karst features such as fractures and cavities. In order to understand the significance of the resistivity values measured in the field, it is important to establish a relationship between ER and/or the formation factor and its potential use in the interpretation of subsurface features. As such, laboratory analyses for physical parameters of soil and rock samples, such as the effective porosity ER (and associated formation factor) were measured. These values, together with the field ER images, were then used to assess the subsurface distribution of porosity and other associated features such as fractures and cavities. The depth to the bedrock was determined by subsurface ER and porosity distribution along each line.

To identify subsurface zones and the depth of each zone, subsurface ER distribution, porosity, and void ratio distributions were determined. The coordinates of each of the data points of the ER images from North-South, East-West, and along the diagonal line were determined. The spatial distribution of limestone and some structures associated with the geotechnical problems in the subsurface were evaluated by using the SURFER (ver.8.2) software to generate the 2D and 3D representations of the subsurface topography of the area.

## 2. Results and Discussion

Porosity measurements on subsurface rock and soil samples obtained from the laboratory work are shown in Table 1. The results show that rock formations that consist of unweathered and weathered limestone have porosities ranging between 0.33% to 24.04%, while the soils porosity is between 43.62 to 50%.

Table 2 presents the laboratory measurements of ER for the rock, soil, and water samples from the study area. The essence of this is to obtain the resistivity formation factor. A relationship between the formation factor and porosity of the integrated soil and rock samples has been established by using the power fitted equation (1).

**Figure 5:**
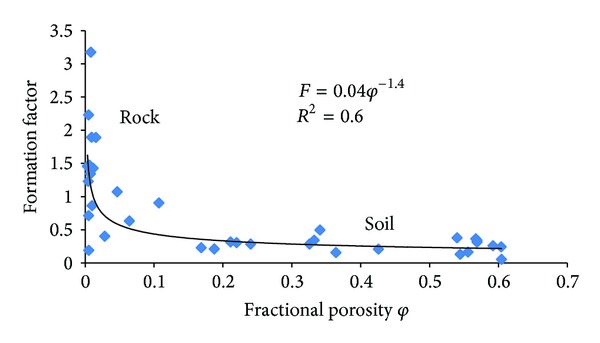
Variation of formation factor against porosity of rock and soil samples.

To relate the formation factor and the fractional porosity of the earth materials (Table 3) found in the study area, the results from the rock and soil measurements were integrated. The purpose of this integration was to obtain a single equation that describes how the formation factor will vary as the fractional porosity of the earth material varies within the study area (Figure 5). This equation was then used to calculate the subsurface porosities indicated by the results of the 2D ER imaging survey performed in the area.

The power fitted regression of data points obtained in the present investigation yields

(2) $$\begin{array}{c} F={0.04\varphi }^{-01.4}\;\text{with:}\;R^{2}=0.6. \end{array}$$

This equation is then applied to calculate the porosity of the subsurface from 2D resistivity imaging using the resistivity modelling software RES2DINV.

### 2.1. Interpretation of 2D ER Imaging

**Figure 6:**
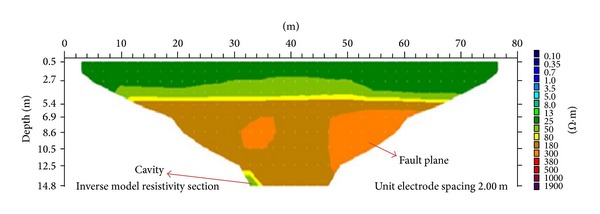
2D resistivity image of first line showing cavity and fault plane.

The first ER profile line (Figure 6) was oriented East-West. The inverse model resistivity section shows a pronounced anomaly at the top of approximately 0.5 to 4.5 m. Stations 0 and 80 m (electrode positions) have resistivity of 25 Ω·m, which falls within the typical resistivity range of a humid soil in the study area. However, at a depth of 4.5 to 5.4 m, the resistivity value was 50 Ω·m, which falls within the alluvium resistivity values. At a depth of 5.4 to 6 m, the resistivity value was found to be 80 Ω·m, indicating a highly weathered limestone layer. Then at the depth of 6 to 14 m, between the horizontal distances of 10 to 68 m, lies a moderately weathered limestone, which is the dominant layer with a resistivity value of 180 (Ω·m). An increase in resistivity of 300 Ω·m can be observed at the centre and the right-hand side of the image at a depth of 6.9 m, which indicates slightly weathered limestone. A fault plane can be seen at the right-hand side of the image towards the centre facing the slightly weathered limestone. At the bottom of the image on the left-hand side is a small portion that shows a decrease in resistivity of 50 Ω·m; this decrease in resistivity suggests the presence of a cavity [20].

**Figure 7:**
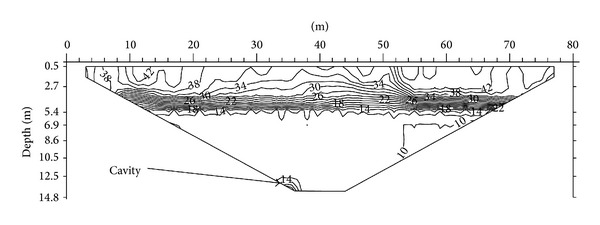
A 2D porosity image of the first profile line indicating cavity affected area.

The porosity along the first line ranges from 10% to 42%.  Figure 7 shows the subsurface porosity distribution of the area. By comparing this with the ER distribution along the same line, slightly weathered limestone with porosity of 10% is found to be in the lower part of the traverse. The humid soil, which has porosity of around 34% to 42%, is situated at the upper part of the image and the alluvium, having a porosity ranging from 16% to 34%, is found beneath the humid soil. Highly weathered limestone with porosity between 14% and 16% is located below the alluvium. The moderately weathered limestone with porosity between 10% and 14% can be seen in between the slightly weathered limestone and the highly weathered limestone. In the lower part of the image lies a small portion that shows higher porosity than the upper layer. This is believed to be a cavity formed by the pressure of the top layer.

**Figure 8:**
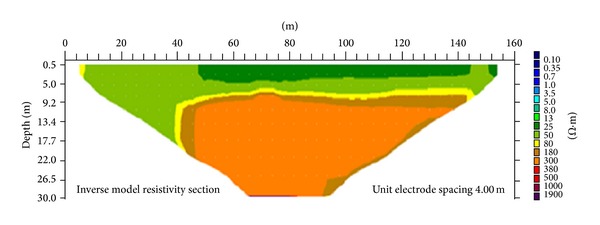
Investigation of 2D resistivity image of second line.

The second ER profile is the diagonal line (Figure 8) with a length of 160 m. This line runs from the northwest to southeast across the survey area. A resistivity value of 25 Ω·m can be found along the profile line between the horizontal distances of 48 to 148 m at a depth of 5 m. This zone is identified as the humid soil. The alluvium zone with resistivity of 50 Ω·m is found to be underlying the humid soil layer. Highly weathered limestone with resistivity of 80 Ω·m forms a very thin layer along the profile line between 40 and 144 m at a depth of 9 m. Below this layer lies a moderately weathered limestone (180 Ω·m). The slightly weathered limestone with a resistivity value of 300 Ω·m can be found at the bottom of the image, at a depth of 20 m, between distances of 40 and 140 m along the profile line (Figure 8).

**Figure 9:**
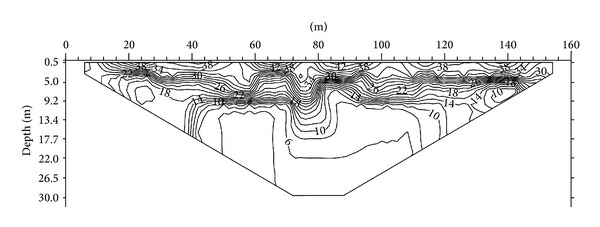
Investigation of 2D porosity image of second line.

Figure 9 shows the subsurface porosity distribution of the diagonal line. By comparing this with the resistivity distribution image of the same line, the porosity can be seen to range from 6% to 46%. The top of the image is humid soil, which has porosity of 34% to 46%. The alluvium has porosity ranging between 20% and 34%. The highly weathered limestone has porosity ranging from 16% to 20%, whereas the moderately weathered limestone has porosity ranging from 10% to 16% and the slightly weathered limestone has porosity of 6% to 10%. In this line, the moderately weathered limestone covers the slightly weathered limestone.

**Figure 10:**
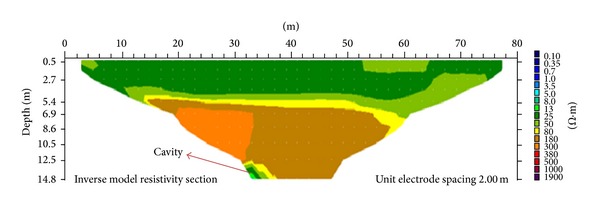
Investigation of 2D resistivity image of third line.

The third ER line is aligned North-South in the field with a length of 80 m. The subsurface resistivity distribution of the area is shown in Figure 10. The resistivity value of 25 Ω·m can be found along the profile line between 0 and 80 m horizontal distance, at vertical depths of between 0.5 and 4 m. This layer is the humid soil and below this zone is the alluvium layer with resistivity of 50 Ω·m. A highly weathered limestone can be found at a vertical depth of 1.5 m below the ground. This layer has resistivity of 80 Ω·m and is continuous throughout the section. A moderately weathered limestone with resistivity of 180 Ω·m can be found between 14 and 58 m horizontal distance, at a depth of 5.4 m. This layer is the uppermost part of the slightly weathered limestone with a resistivity value of 300 Ω·m. An anomaly is found at the bottom of the image on the left-hand side. This anomaly is interpreted as a possible cavity within the limestone. The decrease in resistivity can be associated with the presence of a cavity.

**Figure 11:**
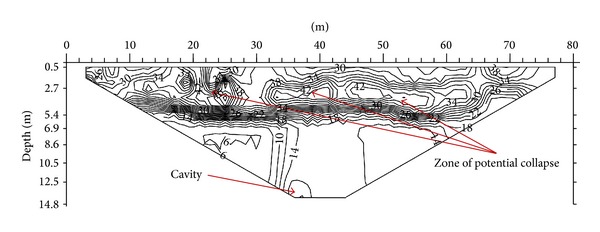
Investigation of 2D porosity image of third line.

Figure 11 shows the subsurface porosity distribution of the third line. By comparing this with the resistivity distribution image of the same line, the porosity can be seen to range from 6% to 46%. The top part of the image is believed to be humid soil, which has porosity from 34% to 46%. This is followed by an alluvium layer with porosity ranging between 20% and 34%. A highly weathered limestone zone with porosity between 16% and 20% can be seen under the alluvium layer. A moderately weathered limestone with porosity ranging from 10% to 16% was found beneath the highly weathered limestone, followed by slightly weathered limestone (porosity 6% to 10%). At the bottommost left-hand side of the image lies a cavity.

### 2.2. Subsurface Topographic Features of the Limestone Obtained in the Study Area

**Figure 12:**
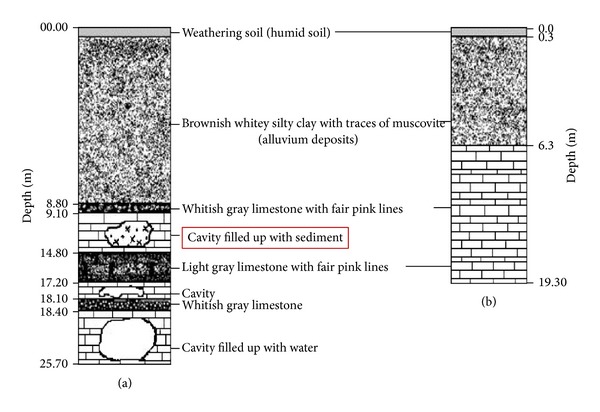
Borehole information in Batu Cave adopted from [20].

This section focuses on the detection of subsurface anomalies within the area, such as sinkholes, cavities, soil pipes, and fractures and then converting them through the use of ER survey techniques into 2D and 3D representations to provide a clear understanding of the entire system and of the geotechnical problems that could be associated with these geologic structures. This was based both on the integration of information from the laboratory resistivity measurement of the soil and rock samples and on the information extracted from the contour lines in the resistivity images. This information is then compared with the borehole information (Figure 12) of the area for validation.

**Figure 13:**
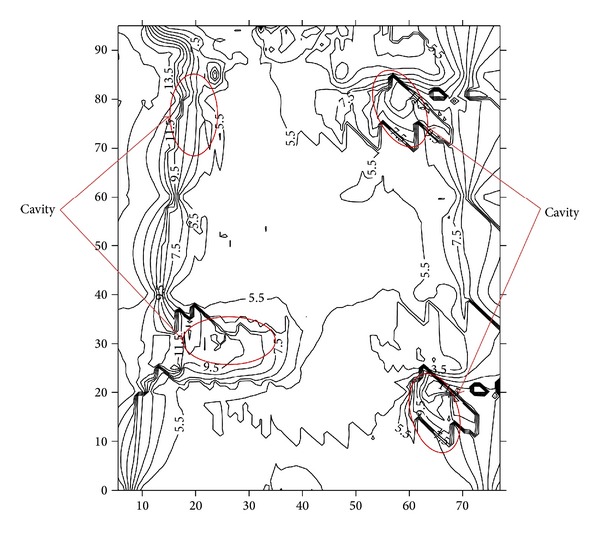
2D representation of the topography of the study area (red circles show the location of the cavities).

Based on the information extracted from all the resistivity and porosity lines of the study area, the distribution of the geohazard-related structures and the topography of the area are shown in Figure 13. The 2D image shows the topography of the study area. This figure, in comparison with all the ER lines in the field, shows that there are some cavities in the subsurface below a depth of 10.5 m. These findings agree with the results of the borehole records and gravity results shown in Figures 12 and 13. The regions enclosed by the red circles in Figures 12 and 13 shows the presence of cavities.

**Figure 14:**
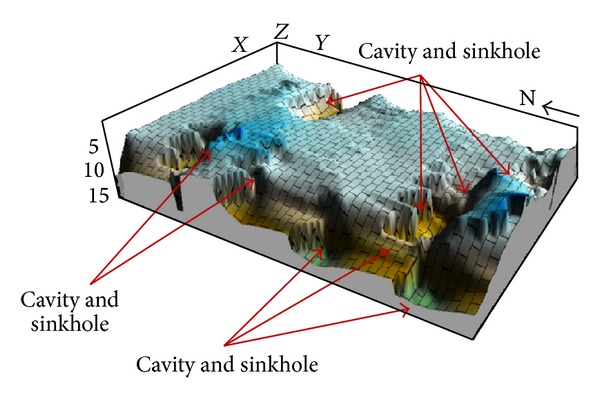
3D representation of subsurface limestone in study area.

Geohazard structures and other related features within the subsurface of the study area are presented in 3D view in Figure 14. This illustrates evidence that clearly shows the presence of cavities and/or sinkholes within the study area. The 3D representation also indicates the magnitude and structural distribution of the cavities and how some cavities or sinkholes (as represented by the deep and/or sharp depressions within the limestone) are presented at a depth of around 9.5 to 14.5 m in some parts of the area.

## 3. Conclusions

The intention of this paper was to develop a geometrical representation of cavities and sinkholes based on 2D and 3D ER techniques, by establishing a relationship between soils and rock samples obtained from laboratory measurements and field ER measurements. The resistivity data obtained from the field surveys were analysed to determine the subsurface boundaries and layers of limestone and other structures. The formation factor *F* = 0.0406*φ* ^−01.402^ and fractional porosity *φ* relationship were then used to calculate the subsurface porosities of the earth materials.

These results were then integrated with the field ER measurements to produce 2D and 3D images of the surface and subsurface structures, respectively. The presence of cavities and sinkholes was determined in the northeast, northwest, southwest, and southern parts of the study area.

This work illustrates how the integration of laboratory and field analysis can assist in creating geohazard maps. The results also give better information of subsurface structural systems based on 3D features. This work would facilitate the ability of engineers and environmental managers to develop reliable sustainable management plans for the prevention of the catastrophic collapse of building structures and other related geohazard disasters.

## Figures and Tables

**Table 1:** Porosity of earth materials from the study area.

Sample material	Location	Porosity %
Soil	Football field	43.62%–50%
Un weathered limestone	Batu Cave Mountain	0.33%–4.66%
Weathered limestone	Batu Cave Mountain	10.7%–24.04%

**Table 2:** Electrical resistivity of earth materials from the study area.

Sampled material	Location	Resistivity (Ω*·*m)
Rainwater/surface water	Football field	7.44–261.99
Spring water	Kg Batu	65.66–151.72
Soil	Football field	26.56–204.54
Un weathered limestone	Batu Cave Mountain	78.40–348.97
Weathered limestone	Batu Cave Mountain	14.31–75.04

**Table 3:** Classification use for the description of rock mass/material in the study area.